# Supplementary material for: Fish Sidestream-Derived Protein Hydrolysates Suppress DSS-Induced Colitis by Modulating Intestinal Inflammation in Mice
Source: Mar Drugs. 2021 May 28;19(6):312. doi: 10.3390/md19060312 (PMC8228426; doi:10.3390/md19060312)
Supplement: Supplementary file 1 [file marinedrugs-19-00312-s001.zip › marinedrugs-1190007-supplementary.pdf]

marine drugs

Supplementary Material for

Fish sidestream-derived protein hydrolysates suppress DSS-induced colitis by modulating intestinal inflammation in mice

Maria G. Daskalaki [1,2], Konstantinos Axarlis [1,2], Tone Aspevik [3], Michail Orfanakis [1,2], Ourania Kolliniati [1,2], Ioanna Lapi [1,2], Maria Tzardi [4], Eirini Dermitzaki [1], Maria Venihaki [1], Katerina Kousoulaki [3] and Christos Tsatsanis [1,2,*]

[1] Laboratory of Clinical Chemistry, Medical School, University of Crete, Heraklion 70013, Greece; m.daskalaki@med.uoc.gr (M.G.D.); orfanakis3012m@gmail.com (M.O.), konax@outlook.com (K.A.); raliakolliniatis21@gmail.com (O.K.); iwanna_lapi@hotmail.com (I.L.); renaderm@med.uoc.gr (E.D.); venihaki@med.uoc.gr (M.V.)
[2] Institute of Molecular Biology and Biotechnology, FORTH, 71100 Heraklion, Greece
[3] Department of Nutrition and Feed Technology, Nofima AS, 5141 Bergen, Norway; Tone.Aspevik@Nofima.no (T.A.), katerina.Kousoulaki@Nofima.no (K.K.)
[4] Laboratory of Pathology, School of Medicine, University of Crete, Heraklion 70013, Greece; tzardi@med.uoc.gr
* Correspondence: tsatsani@uoc.gr, Tel.: +30-2810394833

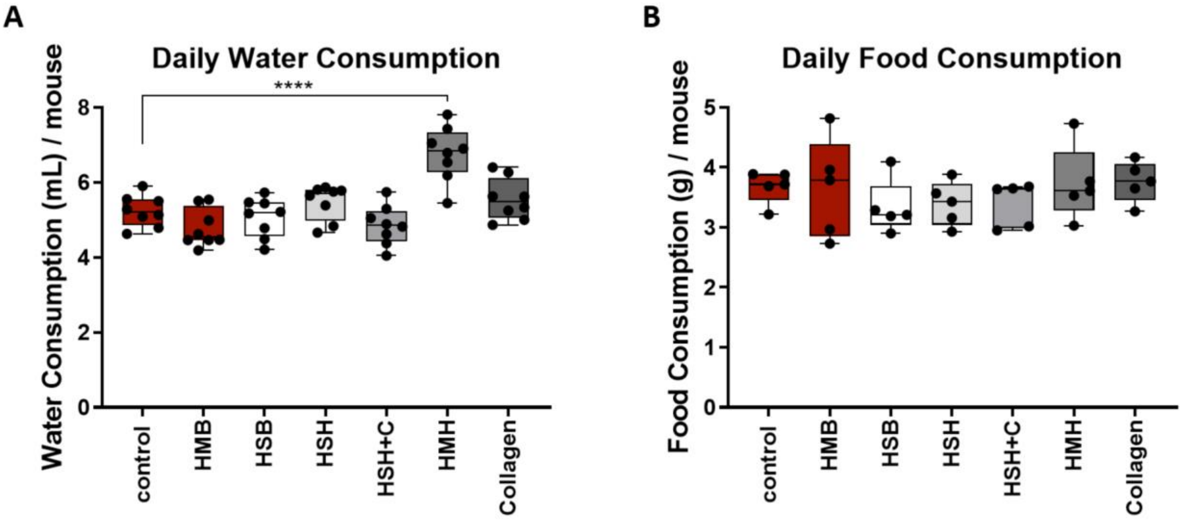

Figure S1. Daily A. water and B. food consumption of animals fed diets containing the different supplements. Graphs represent median ± SD and 2-way ANOVA statistical analysis was performed. ****p<0.0001.

Table S1. Composition of the different fish sidestream-derived supplements used in the cutaneous model.

**Chemical composition**		**HMB**	**HMH**	**HSB**	**HSH**	**Collagen**
Crude protein Kjeldahl (N*6.25)	%	82.4	59.3	89.8	89.3	>90
Total dry matter	%	96.3	96.4	96.3	98	96.7
Ash	%	15.7	38.3	9.3	12.6	3.1
Watersoluble crude protein	g/100g sample	82	58.5	88.7	89.2	>95
**Peptide size distribution**						
Mw-peptide > 20000	% of water sol. peptides	<0.1	<0,.1	<0,.1	<0,.1	<0,.1
Mw-peptide 20000-15000	% of water sol. peptides	<0,.1	<0,.1	<0,.1	<0,.1	<0,.1
Mw-peptide 15000-10000	% of water sol. peptides	0.1	0.1	0.1	0.1	0.1
Mw-peptide 10000-8000	% of water sol. peptides	0.1	0.3	0.2	0.2	0.5
Mw-peptide 8000-6000	% of water sol. peptides	0.5	1	0.9	1	2.4
Mw-peptide 6000-4000	% of water sol. peptides	1.9	3.7	3.3	3.6	9.4
Mw-peptide 4000-2000	% of water sol. peptides	8.2	13.9	12.9	15.9	28.1
Mw-peptide 2000-1000	% of water sol. peptides	14.5	17.7	18.4	22.2	28
Mw-peptide 1000-500	% of water sol. peptides	17.7	16.6	18.9	19	17.6
Mw-peptide 500-200	% of water sol. peptides	19.7	16.9	20.3	17.3	9.3
Mw-peptide 200-	% of water sol. peptides	37.3	29.7	24.9	20.7	4.6
**Total amino acids**						
Aspartic acid	g/100g sample	6.5	4.4	7	6.8	6.4

Glutamic acid	g/100g sample	10.7	7.4	10.9	10.8	10
Hydroksyproline	g/100g sample	1.4	1.9	3	4.1	9.8
Serine	g/100g sample	3.1	2.5	3.4	3.8	6.1
Glycine	g/100g sample	5.3	6.2	8.8	12.1	26
Histidine	g/100g sample	5	1.8	1.8	1.7	1.1
Arginine	g/100g sample	4.5	3.7	5.3	5.6	8.9
Threonine	g/100g sample	2.9	2	3.2	2.9	2.8
Alanine	g/100g sample	4.6	4.2	5.6	6	9.8
Proline	g/100g sample	3	3.1	4.5	6.1	11.7
Tyrosine	g/100g sample	1.7	1.1	1.7	1.4	0.37
Valine	g/100g sample	3.1	2	3.2	2.8	1.9
Methionine	g/100g sample	1.9	1.4	2.3	2.4	2.4
Isoleucine	g/100g sample	2.4	1.5	2.6	2.1	1.1
Leucine	g/100g sample	5	3.2	5	4.3	2.4
Phenylalanine	g/100g sample	1.9	1.5	2.3	2.3	2.1
Lysine	g/100g sample	6.9	4.3	6.5	5.5	3.7
**Quality parameters**						
Putrescine	mg/kg	63	44	110	180	41
Cadaverine	mg/kg	<20	<20	<20	<20	<20
Histamine	mg/kg	74	38	67	68	23
Trimethylamin-N	mg N/100 gram	17	15	14	<1	66
Trimethylaminooxide-N	mg N/100 gram	162	53	125	<1	5
**Free amino acids**						
Aspartic acid	g/100 g sample	0.06	0.07	0.09	0.1	0
Glutamic acid	g/100 g sample	0.39	0.27	0.36	0.32	0.01
Hydroksyproline	g/100 g sample	0.01	0.01	0.02	0.03	0
Serine	g/100 g sample	0.09	0.12	0.12	0.16	0.02

Asparagine	g/100 g sample	0.02	0.01	0.02	0.02	0.01
Glycine	g/100 g sample	0.15	0.21	0.15	0.21	0.05
Glutamine	g/100 g sample	0.21	0.43	0.43	0.45	0
Histidine	g/100 g sample	3.2	0.81	0.22	0.2	0
Threonine	g/100 g sample	0.1	0.1	0.14	0.14	0
Alanine	g/100 g sample	0.29	0.27	0.44	0.4	0.03
Arginine	g/100 g sample	0.25	0.34	0.19	0.21	0.02
Proline	g/100 g sample	0.06	0.09	0.1	0.07	0
Tyrosine	g/100 g sample	0.18	0.26	0.18	0.14	0.02
Valine	g/100 g sample	0.1	0.14	0.23	0.19	0.01
Methionine	g/100 g sample	0.29	0.36	0.47	0.37	0
Cysteine	g/100 g sample	0	0	0	0	0
Isoleucine	g/100 g sample	0.08	0.13	0.24	0.19	0.01
Leucine	g/100 g sample	0.42	0.78	0.83	0.72	0.04
Phenylalanine	g/100 g sample	0.3	0.43	0.48	0.67	0.07
Tryptophane	g/100 g sample	0.07	0.07	0.12	0.1	0
Lysine	g/100 g sample	0.56	0.39	0.4	0.3	0
Creatinine	g/100 g sample	0.94	0.15	0.4	0.34	0.07
β-alanine	g/100 g sample	0	0	0.14	0.21	0
Taurine	g/100 g sample	0.85	1.4	0.46	1.3	0.03
4-aminobutanoic acid	g/100 g sample	0	0	0	0.01	0
Citrulline	g/100 g sample	0	0	0	0.01	0
Carnosine	g/100 g sample	0.06	0.03	0.08	0.03	0.01
Anserine	g/100 g sample	0.11	0.05	2.3	0.64	0
L-Ornithine	g/100 g sample	0.02	0.02	0.02	0.02	0

Table S2. List of oligonucleotides used in real time PCR reactions.

**Primer**	**Sequence**
Actin-Fwd	CATTGCTGACAGGATGCAGAAGG
Actin-Rev	TGCTGGAAGGTGGACAGTGAGG
TNFα-Fwd	GCCCACGTCGTAGCAAACCACC
TNFα-Rev	CGGGGCAGCCTTGTCCCTTG
IL-6-Fwd	CAAAGCCAGAGTCCTTCAGAG
IL-6-Rev	CACTCCTTCTGTGACTCCAGC
IL-1β-Fwd	CGGACCCCAAAAGATGAAGGGCTG
IL-1β- Rev	GCTCTTGTTGATGTGCTGCTGCGAG
Cxcl1- Fwd	CCCAAACCGAAGTCATAGCCA
Cxcl1- Rev	CTCCGTTACTTGGGGACACC
Cxcl2-Fwd	CGCCCAGACAGAAGTCATAGCCAC
Cxcl2-Rev	CGTTGAGGGACAGCAGCCCAG
Ccl2-Fwd	GGCTGGAGCATCCACGTGTTGG
Ccl2-Rev	TTGGGGTCAGCACAGACCTCTCTC
Ccl3-Fwd	GAAGGATACAAGCAGCAGCG
Ccl3-Rev	TTCTCTTAGTCAGGAAAATGACACC
IL-10 Fwd	GCGCTGTCATCGATTTCTCCCCTG
IL-10-Rev	GGCCTTGTAGACACCTTGGTCTTGG
TGF-b-Fwd	GACACACAGTACAGCAAGGTCC
TGF-b-Rev	CGACCCACGTAGTAGACGATG
